# The accuracy and reliability of patient-reported opioid consumption following surgical fracture management

**DOI:** 10.1097/PR9.0000000000001399

**Published:** 2026-02-17

**Authors:** James Bilbrough, Joseph Descallar, Sam Adie, Justine M. Naylor, Ian A. Harris, Deanne E. Jenkin

**Affiliations:** a School of Clinical Medicine, St George & Sutherland Clinical Campuses, Faculty of Medicine & Health, University of New South Wales, Kogarah, New South Wales, Australia; b School of Clinical Medicine, Coffs Harbour Rural Campus, Faculty of Medicine & Health, University of New South Wales, Coffs Harbour, New South Wales, Australia; c Ingham Institute for Applied Medical Research, Liverpool, New South Wales, Australia; d School of Clinical Medicine, The South West Sydney Clinical Campuses, Faculty of Medicine & Health, University of New South Wales, Liverpool, New South Wales, Australia; e St George and Sutherland Centre for Clinical Orthopaedic Research (SCORe), Kogarah, New South Wales, Australia; f Whitlam Orthopaedic Research Centre, Ingham Institute for Applied Medical Research, Liverpool, New South Wales, Australia; g Liverpool Hospital, South Western Sydney Local Health District, Liverpool, New South Wales, Australia

**Keywords:** Self Report, Pain, Postoperative, Analgesics, Opioid, Fractures, Bone, Fracture Fixation, Reproducibility of Results

## Abstract

**Objective:**

Postoperative opioid use is commonly determined by patient-report; however, the accuracy of this measure is unclear. We aimed to determine the accuracy and reliability of patient-reported daily opioid consumption up to day 7 and over weeks 1, 2, and 3 after hospital discharge.

**Methods:**

This was a secondary analysis of a randomised trial which tested the comparative effectiveness of 2 opioid regimens. Participants' daily opioid consumption was recorded by patient self-report and the quantity of returned pills in blister packs during the treatment period. Intraclass correlation coefficient (ICC) and Bland–Altman plots for repeated measures were used to assess the agreement between each collection method.

**Results:**

Records with complete patient-reported daily opioid consumption and returned blister packs were included in the analysis (81 of 120). For day 1 postdischarge, the ICC between patient-reported opioid consumption and consumption determined by blister-pack countback was 0.730. From days 2 to 7, the ICC was consistently greater than 0.8, and the mean difference was consistently less than half a tablet. For week 1 overall, the ICC was 0.904, and the mean difference was −0.4 tablets. For weeks 2 and weeks 3, the ICC was 0.953 and 0.884, respectively.

**Conclusion:**

Patient-reported consumption was a reliable and accurate measure of opioid consumption after hospital discharge. Daily consumption demonstrated less reliability and accuracy during the first 24 hours (day 1) which improved overtime. Although patients tended to under-report use, this difference was not seen as clinically meaningful.

## Abstract 2

Patient-reported consumption was found to be a reliable and accurate measure of opioid consumption after hospital discharge.

## 1. Introduction

Opioids are strong analgesics which have long been used to manage severe acute pain.^27^ Prescribing opioids for chronic pain management is widely contentious given the risks associated with long-term use; however, prescribing has increased over the past decade.^2,3,22^ While a subset of patients suffering with chronic pain have demonstrated some benefit with opioid management, there are high rates of opioid abuse when used long-term, manifesting as dependence, addiction, and overdose.^5,7,29^ The high prevalence of opioid abuse has led to the Centers for Disease Control and Prevention declaring an opioid abuse epidemic, resulting in increased attention to opioid prescription in health care.^3^

Opioids are frequently prescribed by orthopaedic surgeons to effectively manage acute and subacute postoperative pain.^25,33^ As such, orthopaedic patients, particularly trauma patients, are at increased risk of long-term opioid use and abuse.^9,26^ Previous studies investigating the consumption of opioids after orthopaedic surgeries found that most prescribed opioid medications were not consumed.^1,15,19,28^ It is important to have accurate and reliable measures of patient opioid consumption to inform prescribing decisions for postoperative care.^1,15,31^ Most studies investigating postoperative opioid consumption rely on patient self-reported measures, such as patient diaries or surveys.^1,15,19,28,31^ These self-reported measures can be subject to recall biases, intended nondisclosure, and misclassification biases, which may influence overall study validity.^6,8,17^ There is no universal gold standard used to verify the accuracy of these self-reported measures; however, counting leftover pills from patient blister packs is one reliable method.^4^

Few studies have investigated the accuracy and reliability of self-reported opioid consumption in orthopaedic trauma patients. The most recent analysis was by Hijji et al.^10^ who conducted a retrospective review of 241 nonoperative orthopaedic trauma patients and cross-referenced patient surveys with a statewide prescription drug monitoring program. The authors found that 85.5% of patients were accurate at reporting opioid use in the 3 months before their presentation; however, the true amount of opioid consumed or daily changes to consumption over prescription periods were not determined given the study's retrospective nature and administrative data used to confirm patient reports. Furthermore, Hijji et al.^10^ excluded patients who received surgical management and did not disclose the types of orthopaedic injuries in the cohort. Patients discharged following surgical fracture management are an important subset of orthopaedic trauma patients, as they are more likely to be prescribed greater amounts of opioids for longer time periods.^32^ The aims of this study were to determine the accuracy and reliability of patient-reported 7-day daily opioid consumption compared with returned blister-pack quantity following surgical fracture management and over weeks 2 and 3 postdischarge.

## 2. Materials and methods

### 2.1. Study design and participants

The study was nested (planned secondary analysis) within a randomized trial testing the comparative effectiveness of a mild opioid (combination of acetaminophen and codeine) vs strong opioid (oxycodone hydrochloride) for the treatment of postdischarge pain after orthopaedic fracture surgical treatment, with full methods described elsewhere.^12^ The randomized trial involved a trauma cohort where participants were patients admitted to one major trauma hospital in Sydney, Australia, with at least one acute fracture requiring surgical treatment. Patients were included in the study if they had sustained a nonpathological fracture of a long bone (ie, humerus, radius, ulna, femur, tibia, or fibula) or the pelvis, patella, calcaneus, or talus and treated with surgical fixation; were 18 years or older; and were able to comprehend the study protocol, written in English. Patients were excluded if they had known or suspected multisystem trauma injuries (eg, major head, chest, or abdominal injury); known or suspected major infection after surgical treatment; known or suspected opioid dependency; contraindications to study treatment; or were pregnant or breastfeeding. All 120 participants were randomized at the time of hospital discharge into the original trial. For this secondary analysis, we included the 81 participants who completed both self-reported opioid consumption and returned blister packs, which were required for the comparison of reporting accuracy.

### 2.2. Ethical approval and consent to participate

The larger study was approved by Hunter New England Health Human Research Ethics Committee, New South Wales, Australia, Reference: HREC 15/05/20/3.02. Before commencement, the trial protocol was registered on the July 15, 2016, with the Australia New Zealand Clinical Trial Registry No. ACTRN12616000941460. All participants provided written informed consent, and all methods were performed in accordance with the relevant guidelines and regulations. The larger trial's protocol is accessible online doi:10.1001/jamanetworkopen.2021.34988, Supplement 1.

### 2.3. Procedures and data collection

A consecutive series of eligible patients who provided informed consent to participate in a trial provided descriptive data on study entry during hospital admission. These data included age, sex, employment status, diagnosis, type of surgical treatment, mechanism of injury, insurance status, pain, and quality of life. Participants were randomized to 1 of 2 groups at a 1:1 ratio by means of sealed medication package (identical over-encapsulated blinded study packages), with each group receiving 2 different types of postdischarge opioids. Group one was prescribed strong opioids (oxycodone hydrochloride immediate release 5 mg or 10 mg [1 or 2 tablets, respectively]) 4 times/day. Group 2 was prescribed mild opioids (acetaminophen/codeine 500 mg/8 mg or 1000 mg/16 mg [1 or 2 tablets, respectively]) 4 times/day. All participants were prescribed a maximum dose of 8 tablets/day for 14 days (weeks 1 and 2). Prescribed opioids were titrated to caseation between days 15 and 21 (week 3). The study medication was dispensed in 5 blister packs at hospital discharge, with each tablet in its own individual blister. For study weeks 1 and 2, 2 blister packs containing 56 tablets were supplied for each week (total 4 blister packs with 112 tablets). For week 3, 1 blister pack containing 16 tablets was supplied. Participants were permitted to discontinue study treatment after the first 7 days postdischarge (week 1, primary end point) assuming that many may not require pain treatment after this point.

Participant opioid consumption was recorded by 2 collection methods. The first, by patient-report during routine study follow-up visits. Researchers telephoned patients on days 3, 7, 14, and 21 postdischarge and asked each participant to disclose the amount of opioids consumed in the preceding days, among other trial outcomes. The second, by return of prescribed study blister packs once the participants had completed the trial. At the end of the study period, participants were instructed to return their blister packs through prepaid postage packs and researchers counted any remaining pills. An investigator (D.J.) audited 100% of the original trials' hard-copy patient files (self-reported opioid consumption and study medication returns) to ensure the deidentified electronic records held and used for this study were accurate, as this was not the primary focus of the originating trial.

### 2.4. Outcomes

The primary outcomes of this secondary analysis were the accuracy and reliability of patient-reported daily opioid consumption during the first 7 days of treatment after hospital discharge. Secondary outcomes included the accuracy and reliability of patient-reported opioid consumption during weeks 1, 2, and 3 after hospital discharge. This was measured by patient-report through telephone interview (on postdischarge days 3, 7, 14, and 21) and by count of the remaining tablets returned in the study blister pack (gold standard) (of 128 individual blister sealed tablets). We investigated the accuracy of patient-reported opioid consumption compared with return tablets count-back and test–retest reliability between the 2 collection modalities, daily up to day 7 and weekly over the 3-week study period.

### 2.5. Statistical analysis

Intraclass correlation coefficient (ICC) and Bland–Altman plot were used to assess the agreement between the quantity of opioid consumed as measured from patient-reported interviews and from remaining tablets in returned blister packs. The ICC estimates and 95% confidence interval (95% CI) were estimated with mixed-effects models. Mean difference ±1.96 standard deviation (SD) of the bias between both measures were reported from the repeated-measures Bland–Altman analysis. Mann–Whitney *U* test and χ^2^ test were used to assess for differences in baseline characteristics between participants whose blister packs were returned (included) and not returned (excluded). Significance was set at *P* < 0.05, and all statistics were conducted on IBM SPSS Statistics for Windows (version 27; IBM, Armonk, NY) and R version 4.3.3. Sample size was dictated by the original sample; hence, no a priori calculation was performed.

## 3. Results

**Figure 1.**
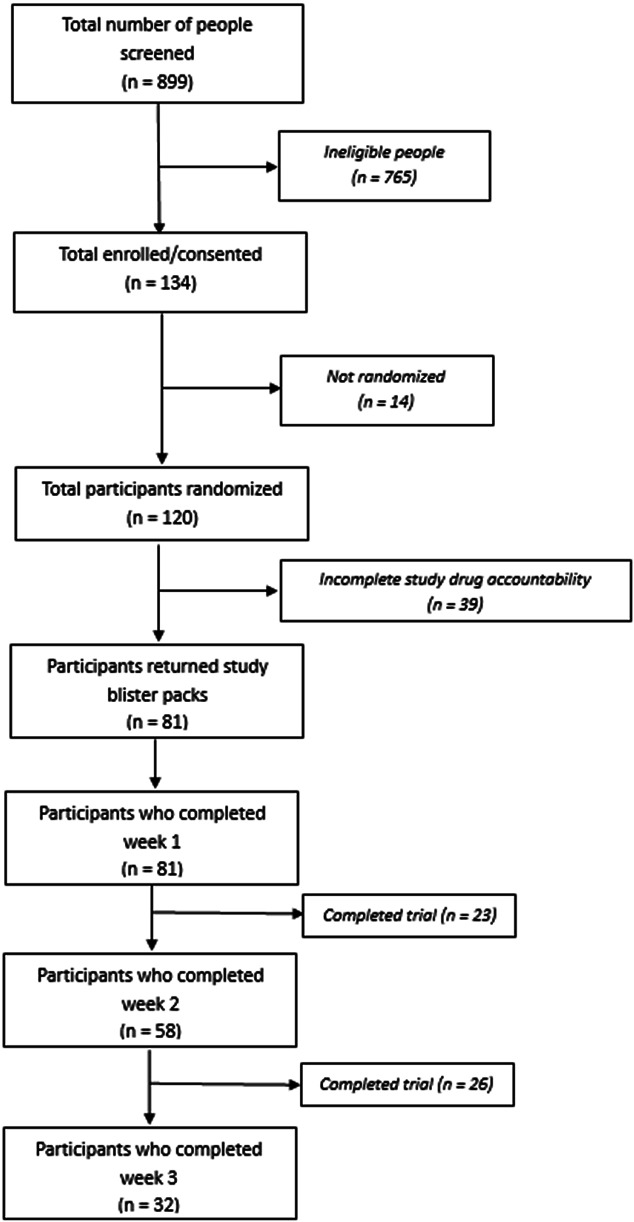
Consort flow diagram of participants through the study.

A consecutive sample of 120 participants met eligibility, gave written informed consent, and underwent orthopaedic surgical fixation for traumatic fractures between July 27, 2016, and August 22, 2017. Records with complete patient-reports of daily opioid consumption and returned blister packs and/or confirmed destruction of empty packs were included in the analysis (81 of 120). Participant flow through to day 7 (ie, primary end point, week 1) and beyond (ie, days 8–21, week 2 and 3) is shown in Figure 1. All 81 participants completed the first week of the study (minimum trial period). Of these, 58 participants (72%) chose to continue into week 2 (days 8–14), and 32 participants (42%) continued into week 3 (days 15–21).

Baseline characteristics of participants who returned blister packs and participants who did not return blister packs at the end of the follow-up period are summarized in Table 1. There was no significant difference in patient characteristics (including age, weight, height, body mass index, length of stay, average pain, and worst pain at discharge) between participants who did and did not return blister packs (*P* > 0.05, Mann–Whitney *U* Test and χ^2^ test). Most participants were male (68%), with a mean age of 39 years (SD, 14.2). There were 71 participants who sustained a single fracture, and most fractures were of the lower limb (60%). Open reduction and internal fixation were the most common surgical management used (86%). The average pain at discharge was 4.2 of 10 (SD, 1.6) and worst pain at discharge was 6.6 of 10 (SD, 1.8).

**Table 1:** Baseline characteristics of participants whose blister packs were returned (included patients) vs not returned (excluded patients).

Baseline characteristics^†^	Participants, n (%)		*P* *
	Blister pack returned (n = 81)	Blister pack not returned (n = 39)	
Sex, n (%)			0.5
Men	57 (68%)	30 (77%)	
Women	24 (32%)	9 (23%)	
Age, mean (SD), y	39 (14.2)	33.7 (12%)	0.07
Weight, mean (SD), kg	89 (24.5)	85.7 (19%)	0.5
Height, mean (SD), cm	173 (14.1)	177 (10%)	0.4
BMI, mean (SD)‡	32 (25.7)	28.4 (7)	0.1
Opioids randomized at discharge			0.4
Strong opioids§	41 (52%)	17 (44%)	
Weak opioids‖	39 (48%)	22 (56%)	
Insurance status, n (%)			0.5
Medicare	26 (3%)	18 (46%)	
Private health insurance	29 (37.2%)	7 (18%)	
Compulsory third party	17 (19%)	10 (26%)	
Worker compensation	7 (9%)	4 (10%)	
Other	2 (2.6%)	0	
Mechanism of injury, n (%)			0.8
Road-related trauma	25 (31%)	12 (30%)	
Fall	29 (36%)	11 (28%)	
Blunt/crush trauma	26 (32%)	15 (39%)	
Other	1 (1%)	1 (3%)	
Employed, n (%)	74 (91%)	32 (82%)	0.7
Nonsmoker^e^	52 (64%)	23 (59%)	0.5
No. of total fractures, n (%)			0.7
One	71 (88%)	35 (90%)	
Two	5 (6%)	3 (7%)	
Three	3 (4%)	0	
Four	2 (3%)	1 (3%)	
Region of fractures, n (%)			0.2
Upper limb¶	24 (25%)	17 (40%)	
Lower limb#	57 (60%)	20 (48%)	
Pelvis	6 (6%)	0	
Other	8 (8%)	5 (12%)	
Fracture management, n (%)			0.4
CRIF	7 (8.6%)	2 (5%)	
OREF	3 (3.7%)	2 (5%)	
ORIF	70 (86%)	35 (90%)	
CREF + OREF	1 (1%)	0	
Length of Hospital stay, mean (SD), days	5.5 (4.2)	5.7 (6.8)	0.05
NRS score, mean (SD)			
Average pain at discharge, mean (SD)	4.2 (1.6)	4 (1.5)	0.8
Worst pain at discharge, mean (SD)	6.6 (1.8)	6.2 (2)	0.2
EQ-5D-5L			
Mobility, n (%)			0.1
No problems	17 (21%)	17 (44%)	
Problems**	64 (79%)	22 (56%)	
Self-care, n (%)			0.4
No problems	5 (6%)	4 (10%)	
Problems**	76 (94%)	35 (90%)	
Usual activity, n (%)			0.4
No problems	0	4 (10%)	
Problems**	81 (100%)	35 (90%)	
Pain, n (%)			0.7
No problems	2 (2%)	1 (3%)	
Problems**	79 (98%)	38 (97%)	
Anxiety and depression, n (%)			0.2
No problems	54 (67%)	22 (56%)	
Problems**	27 (33%)	17 (44%)	
VAS score, mean (SD)	69.4 (13)	67.9 (13.1)	0.7

* *P* value from Mann–Whitney *U* test (continuous data) and χ^2^ test (nominal data).

†Baselined characteristics collected during initial hospital admission for fracture.

‡ BMI was calculated by dividing weight in kilograms by height in meters squared.

§ Strong opioids prescribed were oxycodone hydrochloride immediate release 5 mg or 10 mg (1 or 2 tablets, respectively).

‖ Weak opioids prescribed were acetaminophen/codeine 500 mg/8 mg or 1000 mg/16 mg (1 or 2 tablets, respectively).

¶ Included humerus, radius, and ulnar.

# Included femur, tibia, fibular, patellar, talus, and calcaneus.

** Included levels 2 (mild), 3 (moderate), 4 (severe), and 5 (extreme).

BMI, body mass index; CRIF, closed reduction and internal fixation; OREF, open reduction and external fixation; ORIF, open reduction and internal fixation; CREF, closed reduction and external fixation; NRS, numerical rating score (range, 0–10); EQ-5D-5L, EuroQol5-Dimension 5-Level Questionnaire; VAS, visual analogue scale (range, 0–100).

### 3.1. Daily opioid consumption (days 1–7)

A daily breakdown of patient-reported opioid use compared with opioid use confirmed by blister pack return is summarized in Table 2.

**Table 2:** Patient-reported opioid use vs opioid use confirmed by blister pack from days 1 to 7.

	Patient-reported opioid use	Opioid use confirmed by blister pack
Day 1, mean* (SD)	4.4 (2.6)	6 (2.8)
Day 2, mean (SD)	5.5 (2.9)	5.8 (3)
Day 3, mean (SD)	5 (3.1)	5.5 (3.2)
Day 4, mean (SD)	4.8 (3.2)	5.1 (3.4)
Day 5, mean (SD)	4.4 (3.3)	4.6 (3.5)
Day 6, mean (SD)	4.1 (3.4)	4.3 (3.6)
Day 7, mean (SD)	3.7 (3.4)	4 (3.7)

*Values represent mean tablets; maximum 8 tablets daily; SD = standard deviation; n = 81.

### 3.2. Participation from week 1

From week 1, 76 of 81 participants (94%) returned their first blister pack (days 1–4), and all 81 participants returned their second blister pack (days 5–7). On day 2, one participant did not self-report the quantity of opioids they consumed. On days 3 and 4, one separate participant did not self-report the quantity of opioids they consumed. As such, there were a total of 75 participants included on days 2 to 4, and 81 participants included on days 5 to 7.

### 3.3. Daily opioid consumption (days 1–7, primary outcome)

**Figure 2.**
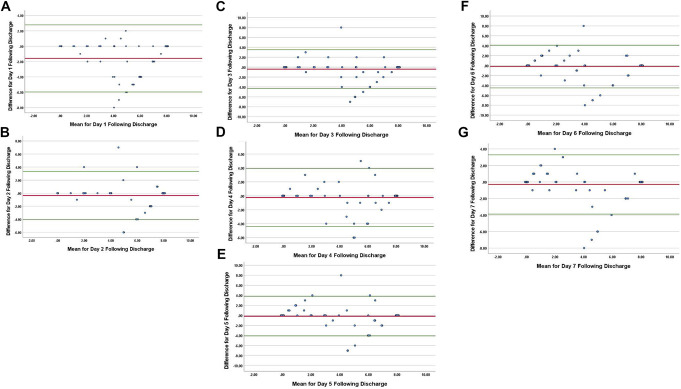
Bland–Altman plots from day 1 to day 7 after discharge. Difference = Blister Pack Count—Self-Reported (# of tablets used). Mean = Average of Blister Pack Count and Self-Reported (# of tablets used). (A) Day 1; Mean −1.59, Standard Deviation (SD) 2.23, Upper Limit of Agreement (ULA) 2.7808, Lower Limit of Agreement (LLA) −5.9608. (B) Day 2; Mean −0.35, SD 1.89, ULA 3.35, LLA −4.05. (C) Day 3; Mean −0.4, SD 2, ULA 3.52, LLA −4.32. (D) Day 4; Mean −0.24, SD 2.12, ULA 3.92, LLA −4.4. (E) Day 5; Mean −0.16, SD 2, ULA 3.76, LLA −4.08. (F) Day 6; Mean −0.2, SD 2.2, ULA 4.111, LLA −4.51. (G) Day 7; Mean −0.3, SD 1.83, ULA 3.29, LLA −3.89.

For day 1, the ICC was 0.730 (95% CI 0.350–0.866). Of the 76 patients, 40 (53%) had identical values between the 2 methods. The mean difference between the number of self-reported tablets consumed and the number determined by blister-pack countback was −1.59 tablets (95% CI −5.96 to 2.78; SD 2.23) (Fig. 2A).

For day 2, the ICC was 0.887 (95% CI 0.821–0.929). Of the 75 patients, 55 (73%) had identical values between the 2 methods. The mean difference between the number of self-reported tablets consumed and the number determined by blister-pack countback was −0.35 tablets (95% CI −4.05 to 3.35; SD 1.89) (Fig. 2B).

For day 3, the ICC was 0.890 (95% CI 0.826–0.932). Of the 75 patients, 54 (72%) had identical values between the 2 methods. The mean difference between the number of self-reported tablets consumed and the number determined by blister-pack countback was −0.4 tablets (95% CI −4.32 to 3.52; SD 1.89) (Fig. 2C).

For day 4, the ICC was 0.886 (95% CI 0.820–0.928). Of the 75 patients, 52 (69%) had identical values between the 2 methods. The mean difference between the number of self-reported tablets consumed and the number determined by blister-pack countback was −0.24 tablets (95% CI −4.4 to 3.92; SD 2.12) (Fig. 2D).

For day 5, the ICC was 0.907 (95% CI 0.855–0.940). Of the 81 patients, 57 (70%) had identical values between the 2 methods. The mean difference between the number of self-reported tablets consumed and the number determined by blister-pack countback was −0.16 tablets (95% CI −4.08 to 3.76; SD 2) (Fig. 2E).

For day 6, the ICC was 0.890 (95% CI 0.829–0.929). Of the 81 patients, 54 (67%) had identical values between the 2 methods. The mean difference between the number of self-reported tablets consumed and the number determined by blister-pack countback was −0.2 tablets (95% CI −4.51 to 4.11; SD 2.2) (Fig. 2F).

For day 7, the ICC was 0.928 (95% CI 0.888–0.954). Of the 81 patients, 55 (68%) had identical values between the 2 methods. The mean difference between the number of self-reported tablets consumed and the number determined by blister-pack countback was −0.3 tablets (95% CI −3.89 to 3.29; SD 1.83) (Fig. 2G).

### 3.4. Weekly opioid consumption (week 1, week 2, and week 3)

A weekly breakdown of patient-reported opioid use compared with opioid use confirmed by blister-pack return is summarized in Table 3.

**Table 3:** Patient-reported opioid use vs opioid use confirmed by blister pack from week 1, week 2, week 3.

	Patient-reported opioid use	Opioid use confirmed by blister pack
Week 1*, mean (SD)	4.5 (3.2)	5 (3.4)
Week 2†, mean (SD)	4.5 (3.4)	4.8 (3.6)
Week 3‡, mean (SD)	1.8 (1.3)	1.7 (1.3)

* Values represent mean tablets; maximum 8 tablets daily; SD = standard deviation; n = 81.

† Values represent mean tablets; maximum 8 tablets daily; SD = standard deviation; n = 58.

‡ Values represent mean tablets; maximum tablets tapered from 4 on day 15 to 1 on day 21; SD = standard deviation; n = 32.

### 3.5. Weekly opioid consumption (weeks 1–3, secondary outcome)

**Figure 3.**
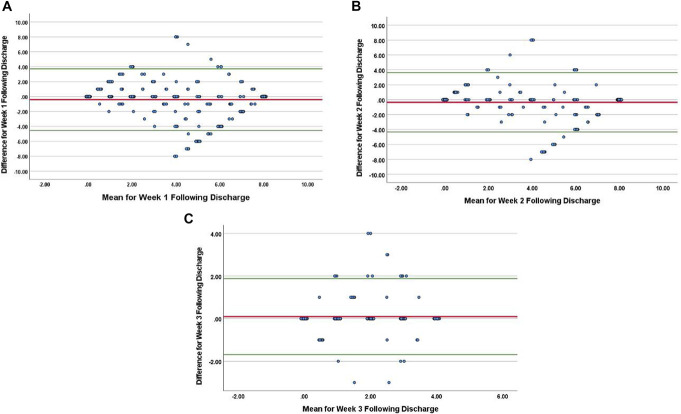
Bland–Altman plots from week 1, week 2, and week 3 after discharge. Difference = Blister Pack Count—Self-Reported (# of tablets used). Mean = Average of Blister Pack Count and Self-Reported (# of tablets used). (A) Week 1; Mean = −0.408, Standard Deviation (SD) = 2.1, Upper Limit of Agreement (ULA) = 3.720, Lower Limit of Agreement (LLA) = −4.536. (B) Week 2; Mean = −0.347, SD = 2, ULA = 3.637, LLA = −4.330. (C) Week 3; Mean = 0.09, SD = 0.91, ULA = 1.877, LLA = −1.688.

For week 1, 544 (96%) of 567 individual daily blisters were returned and the patients returning these had provided self-reported quantities. The ICC between blister-pack count and patient self-reported quantity was 0.904. Of the 544 individual blisters, 367 (67%) matched exactly with the self-reported quantities. The mean difference between the number of self-reported tablets consumed and the number determined by blister-pack countback was −0.408 tablets (95% CI −4.536 to 3.720; SD 2.1) (Fig. 3A).

Of the 81 participants in week 1, 58 participants (72%) continued into week 2 and returned their blister packs. From 406 dispensed individual blisters, there were 378 (93%) which were returned with the associated participants providing self-reported quantities. The ICC between blister pack count and patient self-reported quantity was 0.953. Of the 378 returned blisters, 280 (74%) matched exactly with the self-reported quantities. The mean difference between the number of self-reported tablets consumed and the number determined by blister-pack countback was −0.347 tablets (95% CI −4.330 to 3.637; SD 2) (Fig. 3B).

Of the 58 participants in week 2, 32 participants (55%) continued into week 3 and returned their blister packs. From 224 dispensed individual blisters, there were 185 (83%) which were returned with the associated participants providing self-reported quantities. The ICC between blister-pack count and patient self-reported quantity was 0.884 (95% CI 0.844–0.913). Of the 185 returned blisters, 149 (81%) matched exactly with the self-reported quantities. The mean difference between the number of self-reported tablets consumed and the number determined by blister-pack countback was 0.09 tablets (95% CI −1.688 to 1.877; SD 0.91) (Fig. 3C).

## 4. Discussion

In this secondary analysis of a randomized trial, we found that patient-reported consumption was a reliable and accurate measure of opioid consumption during the first 3 weeks after discharge from surgical fracture treatment when compared with blister-pack countback at the end of the follow-up period. Overall, the test–retest ICC values observed were high. With the exception of day 1, which demonstrated moderate agreement, we found that there was good to excellent agreement between patient-reported opioid consumption and consumption determined by blister-pack countback over the 3 week study period.^18^ The associated confidence intervals of the ICC values were consistently narrow, allowing us to be reasonably confident that the true reliability is good to excellent. More than half of the participants reported exact matches on day 1, and this proportion increased over the study's duration. The mean difference determined by Bland–Altman analysis suggested that patients had a tendency to under-report their opioid consumption, although this was on average less than half a tablet (of a possible 8 tablets daily) on all days in week 1 (except day 1) and weeks 2 and 3. Moreover, the confidence intervals and standard deviations of the mean difference indicate that there was some variability in patient-reported consumption, which improved beyond day 1 and differences were not significant. Day 1 after discharge was found to have the lowest ICC with poor confidence, and the largest difference between patient-reported opioid consumption and consumption determined by blister-pack countback. We postulate that a significant proportion of patients experienced poor recall memory during the acute postoperative period, which proceeded to improve during the first week.^30^ Furthermore, cognitive overload—secondary to simultaneously managing discharge instructions, mobility issues, postoperative pain, and the emotional and environmental transition from hospital to home—may have further contributed to poor recall on day 1.

To the best of our knowledge, this is the first study to assess daily patient-reported opioid consumption with a control measure (blister-pack countback) in a subacute postsurgery population. Hijji et al.^10^ conducted a retrospective analysis of 241 nonoperative orthopaedic patients and found that 85.5% of patients were accurate at reporting consumption when compared with a statewide prescription monitoring database. However, given that study's retrospective nature and their implementation of a prescription monitoring database as the comparative gold standard, it was unclear how much opioid was actually consumed or what the daily changes were in accuracy of reporting. Similar to our findings, Daoust et al.^4^ reported an overall excellent agreement between self-reported opioid consumption and consumption confirmed by prescription bottle countback in emergency department discharge patients during a 14-day follow-up. However, a limitation of that study was that the authors did not analyse daily agreement and reliability. We found daily ICC and Bland–Altmann analysis over the first week to be a valuable metric, as it demonstrated that patients were less accurate at self-reporting medication consumption at the start of the study period. Lacasse et al.^20^ reported lower accuracy with patient-reported opioid consumption compared with a prescription claims database in patients with chronic pain compared with what we observed in our acute pain population.

Our findings align with previous studies assessing opioid consumption in patients with acute pain, where self-reported measures demonstrated high reliability and minimal clinically significant under-reporting.^6,8,14,21^ However, this contrasts with studies involving patients with chronic pain,^4,20^ where self-reported opioid use has been shown to be less accurate, often due to social desirability bias and inconsistent recall.^20^ For example, Lacasse et al.^20^ found lower agreement between self-reported opioid use and prescription claims data in chronic pain populations, suggesting that long-term opioid users may be more prone to misreporting. These findings are supported by a cross-sectional analysis from Kipping et al.^16^ who demonstrated that chronic pain patients significantly under-reported opioid consumption when compared with preoperative hospital inpatients, and several earlier studies.^11,13,23,24^ This discrepancy highlights the importance of contextualizing self-report reliability within the pain trajectory—acute vs chronic. Furthermore, while our study excluded patients with known or suspected opioid use disorder (OUD), research examining self-report accuracy in this population indicates even greater challenges. Studies have shown that individuals with OUD frequently under-report use due to stigma, fear of repercussions, or impaired recall.^6,14,21^ These contrasts underscore the relative robustness of self-reporting in acute postoperative settings and suggest that findings from such populations may not be generalizable to chronic pain or OUD cohorts; however, it is important to recognise that recall bias may occur in the first day postdischarge.

The strengths of this study include the use of blister packs (gold standard) as a control, the study design ensuring a controlled sample (randomized control trial), providing daily breakdown during week 1 (rather than an individual ICC measurement for the study period), and residual follow-up for 2 additional weeks (until week 3). Nevertheless, there were limitations to this study. As our methodology relied on patient self-report through telephone, there is the potential of a Hawthorne effect, whereby participants increased their reporting accuracy because they were being monitored. As such our findings may not be generalizable to passive, noncontact monitoring scenarios. Although blister-pack countback is considered a reliable method for measuring medication compliance, it is possible that patients may discard tablets rather than consume them, potentially leading to overestimation of adherence. Between days 1 to 4, there were 5 participants who did not return their blister packs and as such were excluded. On days 2, 3, and 4, one participant did not provide self-reported opioid consumption, which meant they were also excluded. Ideally all participants would have continued into week 2 and week 3 to maximise sample size; however, it is important to note that the primary outcome of this study was limited to the first 7 days postdischarge, a period during which all 81 participants completed follow-up. This time frame was chosen based on the original randomized trial design, which permitted cessation of study medication after day 7, anticipating that many patients would no longer require opioids. As such, the attrition observed in weeks 2 and 3 was expected and does not affect the validity of the primary outcome. While secondary outcomes extended into weeks 2 and 3, these were exploratory in nature and interpreted with appropriate caution. Importantly, this study did not include a third arm that captured patient-reported opioid consumption without human interaction—such as through diaries, mobile applications, or sensor-enabled packaging. Including a passive-reporting group in future research may help delineate the influence of human interaction on recall and reporting accuracy. However, the clinical utility of such methods remains uncertain, particularly in contexts where opioids—classified as drugs of addiction—are typically prescribed and monitored through in-person clinical consultation. Furthermore, external validity is limited as this was a single-centre study which analysed postoperative orthopaedic trauma patients, as such our findings may not be replicable in other population groups. Although our study included both strong and weak opioid groups, it was not designed or powered to detect differences in compliance or attrition between these subgroups. Future research may consider exploring these comparisons to better understand how opioid strength influences reporting accuracy and adherence.

Recommendations based on our findings highlight the value of patient self-reporting as a reliable and cost-effective method for monitoring opioid consumption after hospital discharge or in community settings. This approach has the potential to inform safer prescribing practices, reduce excess opioid supply in the community, and streamline follow-up protocols. Our study demonstrates patient self-reported opioid consumption is an accurate measure for recording postoperative opioid consumption, especially when compared with the financial, logistical, and environmental burden of organising blister packs and their return. It is important to note that in the acute period postdischarge (ie, the first 24 hours), participants may be subject to recall bias and prescription tablet countback is recommended as the gold standard.

## 5. Conclusion

This secondary analysis of a randomized trial found that patient-reported consumption was a reliable and accurate measure of opioid consumption during the first 3 weeks after hospital discharge. Daily patient-reported consumption during days 1 to 7 demonstrated less reliability and accuracy during the first 24 hours from hospital discharge (day 1) which improved throughout the first week (up to day 7). Although patients tended to under-report use, this difference was not seen as clinically meaningful. These findings support self-reporting in supervised settings and of controlled drugs. Consideration of our findings during study and trial design where opioid utilization is an outcome is recommended. Future research may be needed to assess generalizability in the absence of human interaction.

## Disclosures

The authors have no conflict of interest to declare.
